# Supplementary material for: Systemic Lipid Metabolism Dysregulation as a Possible Driving Force of Fracture Non-Unions?
Source: Bioengineering (Basel). 2024 Nov 11;11(11):1135. doi: 10.3390/bioengineering11111135 (PMC11592249; doi:10.3390/bioengineering11111135)
Supplement: Supplementary file 1 [file bioengineering-11-01135-s001.zip › bioengineering-3293237-supplementary.pdf]

Supplementary Table S1. Tentative identification of the top VIPs obtained by LC/MS analysis

Category	Name / Formula	Experimental mass	Adduct	Formula
Fatty Acyls	Glutinic acid	129.0172	M+H	C5H4O4
Glycerolipids	TG(62:5)	979.9189	M+H	C65H118O5
Glycerophospholipids	LPC(20:3)	546.3562	M+H	C28H52NO7P
Glycerophospholipids	PHOHA-PC<br>OG-PC	634.3738	M-H	C31H58NO10P
Glycerophospholipids	PC(20:4)	556.3013	M-H	C28H48NO8P
Glycerophospholipids	PS(2-OMe,13Me-14:0/0:0)<br>PS(2-OMe-15:0/0:0)	498.2903	M-H	C22H46NO9P
Glycerophospholipids	PS(2-OMe-19:0/0:0)	554.3469	M-H	C26H54NO9P
Nucleosides, nucleotides, and analogues	IDP	429.0245	M+H	C10H14N4O11P2
Organic acids and derivatives	N2-Fructopyranosylarginine	337.1675	M+H	C12H24N4O7
Organic acids and derivatives	Pyrocatechol sulfate<br>Hydroquinone sulfate	188.9862	M-H	C6H6O5S
Organic acids and derivatives / Organoheterocyclic compounds	Pyroglutamic acid<br>L-beta-Ethynylserine<br>Pyrroline hydroxycarboxylic acid<br>N-Acryloylglycine<br>Dimethadione<br>4-Oxoproline	130.0507	M+H	C5H7NO3
Organoheterocyclic compound	5-Methylphenazine-1-carboxylate	238.0773	M-H	C14H11N2O2
Organoheterocyclic compounds	2,5-Furandicarboxylic acid	157.0112	M+H	C6H4O5
Organic oxygen compounds	Lucuminamide	444.1452	M-H	C19H27NO11
Sterol lipids / Fatty Acyls	C21H32O4	347.2209	M-H	C21H32O4
Sterol lipids	Chenodeoxycholic acid glycine conjugate<br>Glycoursodeoxycholic acid<br>Glycodeoxycholic acid<br>Glycochenodeoxycholic acid	448.3074	M-H	C26H43NO5
Sterol lipids	Tauroursodeoxycholic acid<br>Taurochenodeoxycholic acid<br>Taurodeoxycholic acid<br>Taurohyodeoxycholic Acid (THDCA)	498.2903	M-H	C26H45NO6S
Sterol lipids	Taurolithocholic acid 3-glucuronide	658.3352	M-H	C32H53NO11S

Supplementary Table S2. Classification of the top VIPs obtained by GC/MS analysis

Category	Name	Average MW	Formula
Fatty Acyls	Linoleic acid	280.4455	C18H32O2
Fatty Acyls	Trans-13-octadecenoic acid	282.468	C18H34O2
Organic acids and derivatives	Iminodiacetic acid	133.1027	C4H7NO4
Organic acids and derivatives	Citric acid	192.1235	C6H8O7
Organic acids and derivatives	4-Aminobutanoic acid	103.1198	C4H9NO2
Organic acids and derivatives	2-Aminoadipic acid	161.1558	C6H11NO4
Organic acids and derivatives	Aspartic acid	133.1027	C4H7NO4
Organic acids and derivatives	2-Aminomalonic acid	119.0761	C3H5NO4
Organic acids and derivatives	L-proline	115.1305	C5H9NO2
Organic acids and derivatives	2-Aminooctanoic acid	159.2261	C8H17NO2
Organic acids and derivatives	L-Serine	105.0926	C3H7NO3
Organic oxygen compounds	2,3-butanediol	90.121	C4H10O2
Organic acids and derivatives	3-hydroxybutyric acid	104.1045	C4H8O3
Phenylpropanoids and polyketides	3-hydroxyflavone	238.2381	C15H10O3
